# Racial or ethnic differences on treatment adherence and persistence among patients with inflammatory bowel diseases initiated with biologic therapies

**DOI:** 10.1186/s12876-022-02560-y

**Published:** 2022-12-29

**Authors:** Qian Cai, Zhijie Ding, Alex Z. Fu, Aarti A. Patel

**Affiliations:** 1 Janssen Scientific Affairs, LLC, 1125 Trenton Harbourton Rd, Titusville, NJ 08560 USA; 2 Janssen Scientific Affairs, LLC, 800 Ridgeview Drive, Horsham, PA 19044 USA; 3 Georgetown University Medical Center, 3900 Reservoir Road, Washington, DC 20057 USA

**Keywords:** Crohn’s disease, Ulcerative colitis, Biologics, Adherence, Persistence

## Abstract

**Background:**

Inflammatory bowel disease (IBD) is a chronic disease with the potential for significant morbidity in case of suboptimal treatment (e.g. low treatment adherence). In spite of immense research in IBD, literature on association of IBD with race/ethnicity is fragmented. In this study, we aimed to evaluate the association between race/ethnicity and treatment adherence and persistence among patients with Crohn’s disease (CD) or ulcerative colitis (UC) initiated with biologic therapies.

**Methods:**

This observational, retrospective study utilized the Optum Clinformatics (Optum) Extended Data Mart Socioeconomic Status (SES) database. Adult patients with ≥ 2 medical claims for CD or UC diagnosis, ≥ 1 medical or pharmacy claim for corresponding FDA-approved biologic therapy, and a ≥ 12-month pre-index (index date: date of the first biologic medical/pharmacy claim) continuous health plan enrollment were included. Treatment adherence was measured as the proportion of days covered of ≥ 80% and treatment persistence by the number of days from the index date to the biologics discontinuation date. Switching among biologics was allowed for both treatment adherence and treatment persistence. Multivariable regression analyses were performed to evaluate the association between race/ethnicity and treatment adherence/persistence.

**Results:**

Among patients with CD (*N* = 1430) and UC (*N* = 1059) included, majority were White (CD: 80.3%, UC: 78.3%), followed by African Americans (AA; CD: 10.5%, UC: 9.7%). Among patients with CD, AA were significantly less likely to adhere to biologics (adjusted OR [95%CI]: 0.61 [0.38; 0.99]) and more likely to discontinue biologics earlier (adjusted HR [95%CI]: 1.52 [1.16; 2.0]) during the follow-up period compared to Whites, after adjusting for other patient sociodemographic and clinical characteristics. Among patients with UC, no significant differences in the treatment adherence/persistence were observed between different races/ethnicities.

**Conclusions:**

Patients with CD were found to display racial differences in the treatment adherence and persistence of biologics, with significantly lower adherence and earlier discontinuation in AA compared to Whites. Such differences were not observed in patients with UC. Future studies are warranted to understand the possible reasons for racial differences, particularly in patients with CD.

## Introduction

Crohn's disease (CD) and ulcerative colitis (UC) are the two main subtypes of inflammatory bowel disease (IBD) [1], which are chronic, debilitating, and cannot be cured [2]. In CD, any portion of the gastrointestinal tract can be affected with a transmural inflammation in all the tissue layers of the gastrointestinal lining, whereas in UC, the disease is limited to the mucosa of the rectum and the colon [3]. It can be difficult to distinguish between CD and UC, and the evaluation of the clinical profile including disease symptoms and location, radiological and endoscopic findings, and the histopathology profile may facilitate in differentiating between CD and UC [2, 3].

There has been an increasing trend in the number of patients with IBD (either CD or UC) in the US, with nearly 70,000 new individuals diagnosed every year; by 2014, an estimated 1.6 million adults were affected [4]. Several factors are associated with rising incidence of IBD including smoking, and a diet high in sugar and animal fat and low in fiber and fruits [5]. Traditionally, the prevalence of IBD has been higher among Whites as compared with other races [6–9], however, many of these studies were conducted in areas with low populations of minorities [9–11]. An increase in the incidence and prevalence of IBD among minorities including African Americans (AA), Hispanics, and Asians has been reported [9, 12], and this rise has been rapid especially among AA over the past three decades [9, 13]. There have been differences in the disease extent among different races, for example, peri-anal and fistulizing disease phenotypes of CD are typically more aggressive in AA patients versus others [9, 14–17]. 

In general, literature describing IBD among AA and other races are limited [18]. Racial and ethnic minorities continue to be under-represented in IBD clinical trials. As reported in 2020, AA and Hispanics comprised 12.4% and 18.7% of the US population [19], respectively, however, only 1.9% and 2.3% of IBD trial participants were AA and Hispanics/Latino, respectively [20]. Given the lack of patient heterogeneity in randomized controlled trials (RCTs), real-world studies that include race and ethnicity data can better assess the differences among minority populations.

Biologics are the recommended agents for the treatment of patients with moderate-to-severe CD [21] or UC [22] who are non-responsive to conventional therapies. In chronic diseases such as IBD, treatment required is to be taken lifelong or for a major portion of life, and hence, medication adherence and persistence are significant challenges [23]. Treatment non-adherence or discontinuation is associated with several complications including worsening of disease or relapse, and increased morbidity, mortality, and healthcare cost [23–25]. In a systematic review, Khan et al. evaluated the real-world evidence on the adherence and persistence of biologic therapies in patients with IBD and reported non-adherence to biologics in 38%‐77% of patients, depending on the medication possession rate threshold of < 80% or < 86%. The discontinuation of biologics was reported in 0%-25% of the patients within 3 months in 6 studies and 7%-65% of patients within 12 months in 13 studies [25].

Access to disease-modifying agents for the treatment of immunologic conditions has been shown to vary by demographics, potentially contributing to poorer outcomes and more severe disease in racial/ethnic minorities [26, 27]. A systematic review has also demonstrated the role of race- and socioeconomic status (SES)-related factors in the healthcare delivery and effectiveness of treatment in patients with IBD [18].

For these reasons, understanding the roles of race, ethnicity, and other social determinants of health on treatment adherence is critical. Limited real-world evidence is available in the literature regarding the impact of race, ethnicity, and other SES determinants on treatment adherence among patients with IBD. Hence, the present study aimed to evaluate the association between race/ethnicity and treatment adherence and treatment persistence with the adjustment of other SES and clinical characteristics.

## Methods

### Data source

In this observational, retrospective database analysis, Optum Clinformatics (Optum) Extended Data Mart SES, a de-identified database of administrative claims by members of a commercial insurance plan and Medicare Advantage was utilized. The Optum claims database includes details on patient demographics, enrollment start and end dates, de-identified adjudicated pharmacy claims (e.g., outpatient prescriptions) and medical claims (e.g., inpatient and outpatient services), healthcare facilities, and pharmacies featuring information on physician visit, medical procedure, hospitalization, drug dispensed, date of service/prescription, and the number of days of medication supplied. In addition, the Optum SES database includes SES measures for each member, along with race/ethnicity, education, and household income. In this study, patient confidentiality was preserved, and the anonymity of the patient data was safeguarded. No waiver of informed consent was required from an institutional review board.

### Study design and patient population

**Fig. 1:**
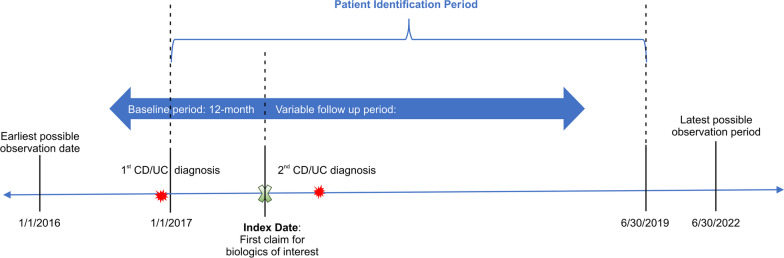
Study design. CD, Crohn’s disease; UC, ulcerative colitis

Study patients were required to have at least one medical or pharmacy claim for FDA-approved biologic therapy, including adalimumab, certolizumab pegol (CD only), golimumab (UC only), infliximab, natalizumab (CD only), vedolizumab, and ustekinumab. The date of the first medical or pharmacy claim for the biologic therapy was designated as the index date. Patients were also required to be aged ≥ 18 years at the index date, having at least 2 medical claims for CD or UC diagnosis on different days from 1/1/2016 to 06/30/2020, with the first diagnosis occurring during the 12-month pre-index period, and having at least a ≥ 12-month pre-index continuous health plan enrollment. To evaluate treatment adherence within the timeframe of interest, patients were required to have at least a 3-, 6-, 9- or 12-month post-index continuous health plan enrollment, respectively. To evaluate treatment persistence over time, a variable follow-up period was adopted. Patients who received any biologic therapy of interest during the 12-month pre-index period or had ≥ 2 different biologic therapies of interest on the index date, were excluded. For patients included in the CD cohort, those with ≥ 1 UC diagnosis at the baseline were excluded; similarly, patients with ≥ 1 CD diagnosis at the baseline were excluded from the UC cohort. Patients with a missing value for race/ethnicity were also excluded (Fig. 1).

### Study variables

The SES characteristics included race/ethnicity, age, gender, region, education level, household income, housing (homeowner/not), and health insurance type (health maintenance organization [HMO], preferred provider organization [PPO], other). HMO is an entity that provides, offers, or arranges for coverage of designated health services needed by members for a fixed, prepaid premium. Members of an HMO generally are required to use participating or approved providers for all health services. PPO is a managed care delivery model consisting of preferred networks of providers with some out-of- network coverage. PPOs offer patients more choice and flexibility than HMOs with correspondingly higher premiums. Whereas Point-of-Service (POS) Plan is a managed care delivery model that combines aspects of an HMO and a PPO. Patients can receive care either from physicians contracted or not contracted through the plan; financial incentives exist for patients to use contracted providers. The clinical characteristics included the Quan-Charlson comorbidity index (QCI), list of comorbidities, immune-mediated inflammatory diseases, surgery, and medication use such as opioids, antibiotics, 5-aminosalicylates, corticosteroids, and immunomodulators, measured during the 12-month pre-index period.

Both pharmacy and medical claims were used to evaluate treatment adherence and persistence for biologics. Values in the days’ supply field for biologics from pharmacy claims were used directly, whereas values of days’ supply for biologics from medical claims were imputed based on the indicated days’ supply of the maintenance dosing schedule for each biologic therapy according to the prescribing information in the US [28–34]. The treatment adherence to biologics was measured as the proportion of days covered (PDC) during the 3-, 6-, 9- or 12-month post-index follow -up period for patients with at least a 3-, 6-, 9- or 12-month continuous enrollment in follow-up, respectively. The PDC was calculated as the sum of the non-overlapped days’ supplies of study treatments (i.e., the number of days the patient is covered by the treatment) divided by days of the follow-up period. Switching to other approved biologics for the treatment of the condition of interest during the follow-up period was considered as a continuation of these biologic therapies. Patients were considered adherent to biologic therapies if PDC ≥ 80%; PDC < 80% was considered non-adherence.

The treatment persistence of biologics was measured by the number of days from the index date to the discontinuation date of the biologic of interest. Similarly, switching to another biologic that was approved for the treatment of the condition during the follow-up period was considered a continuation of these biologic therapies. Discontinuation of the biologic was defined as a gap of larger than 90 days between the end of days’ supply of one prescription and the date of the next prescription; the discontinuation date of the biologic was designated as the fill date plus days’ supply of the last prescription filled prior to the gap in therapy. If discontinuation was not observed, patients were censored at the end of their continuous enrollment or the end of the study period, whichever comes first. The percentage of patients who discontinued their biologic treatment during the follow-up period was described.

### Statistical analyses

Demographics, clinical characteristics, and treatment patterns were examined descriptively. Means and standard deviations were reported for continuous variables whereas numbers and percentages were reported for categorical variables. The time-to-discontinuation among comparison groups were evaluated using Kaplan–Meier curves with log-rank tests.

For multivariate analyses, stepwise and penalized regression with least absolute shrinkage and selection operator (LASSO) methods were used to perform the initial selection of covariates included in regression models. The final decision regarding the inclusion of covariates were made after the review of the results from descriptive analyses, stepwise and penalized regressions. All multivariate analyses were performed among patients with non-missing household income. To evaluate the association between race/ethnicity and 12-month treatment adherence (i.e., PDC ≥ 0.8 vs PDC < 0.8) after adjusting for baseline covariates, multivariable logistic regression was performed among patients with at least a 12-month post-index continuous enrollment. Cox proportional hazard model was conducted to evaluate the association between race/ethnicity and the time to treatment discontinuation after adjusting for baseline covariates including SES and clinical characteristics of interest. All statistical analyses were performed using the statistical analysis software (SAS) Enterprise Guide, version 7.1 (SAS Institute, Cary, NC).

## Results

Overall, 1430 patients with CD and 1059 patients with UC met patient identification criteria and were analyzed.

### Patients with CD

#### Socioeconomic status and clinical characteristics

Of the 1430 CD patients, almost half (50.2%) were male, and the mean (SD) age was 41.8 (16.4) years. The majority of patients were White (80.3%), followed by AA (10.5%), Hispanic (6.1%), and Asian (3.1%). Nearly two-thirds (68.0%) of the AA patients resided in the South region (Virginia, North Carolina, South Carolina, Georgia, Alabama, Mississippi), with low education (only 13.3% had a Bachelor’s degree and above) and low household income (32% had < $40 k income). The mean (SD) QCI was 0.6 (1.2), and the most frequently observed comorbid condition was abdominal pain (63.7%), followed by diarrhea (46.2%) and anemia (32.5%) (Table 1).

**Table 1:** Socioeconomic Status and clinical characteristics of patients with CD or UC

	**CD (*N* = 1430)**				**UC (*N* = 1059)**			
**Parameters**	**White**	**African American**	**Hispanic**	**Asian**	**White**	**African American**	**Hispanic**	**Asian**
**N (%)**	1149 (80.3)	150 (10.5)	87 (6.1)	44 (3.1)	829 (78.3)	103 (9.7)	95 (9.0)	32 (3.0)
Age at index (mean, SD)	42.1 (16.6)	42.8 (17.5)	40.2 (14.5)	35.7 (12.1)	45.5 (17.4)	44.6 (16.8)	40.8 (16.9)	38.7 (13.9)
**Gender, n (%)**								
Male	572 (49.8)	75 (50.0)	45 (51.7)	26 (59.1)	438 (52.8)	50 (48.5)	54 (56.8)	21 (65.6)
Female	577 (50.2)	75 (50.0)	42 (48.3)	18 (40.9)	391 (47.2)	53 (51.5)	41 (43.2)	11 (34.4)
**Region, n (%)**								
Northeast	113 (9.8)	8 (5.3)	6 (6.9)	4 (9.1)	92 (11.1)	3 (2.9)	4 (4.2)	5 (15.6)
Midwest	431 (37.5)	29 (19.3)	11 (12.6)	10 (22.7)	239 (28.8)	15 (14.6)	7 (7.4)	7 (21.9)
South	409 (35.6)	102 (68.0)	42 (48.3)	14 (31.8)	346 (41.7)	78 (75.7)	56 (58.9)	6 (18.8)
West	195 (17.0)	10 (6.7)	28 (32.2)	14 (31.8)	148 (17.9)	6 (5.8)	28 (29.5)	12 (37.5)
Unknown	1 (0.1)	1 (0.7)	0 (0.0)	2 (4.5)	4 (0.5)	1 (1.0)	0 (0.0)	2 (6.3)
**Education level, n (%)**								
< High school	165 (14.4)	56 (37.3)	24 (27.6)	4 (9.1)	125 (15.1)	35 (34.0)	21 (22.1)	1 (3.1)
< Bachelors	662 (57.6)	74 (49.3)	49 (56.3)	15 (34.1)	471 (56.8)	58 (56.3)	57 (60.0)	13 (40.6)
Bachelors +	320 (27.9)	20 (13.3)	14 (16.1)	25 (56.8)	230 (27.7)	10 (9.7)	16 (16.8)	18 (56.3)
Unknown	2 (0.2)	0 (0.0)	0 (0.0)	0 (0.0)	3 (0.4)	0 (0.0)	1 (1.1)	0 (0.0)
**Household income, n (%)**								
< $40 K	151 (13.1)	48 (32.0)	15 (17.2)	4 (9.1)	85 (10.3)	24 (23.3)	15 (15.8)	2 (6.3)
$40-49 K	55 (4.8)	16 (10.7)	12 (13.8)	1 (2.3)	33 (4.0)	8 (7.8)	7 (7.4)	3 (9.4)
$50-59 K	66 (5.7)	18 (12.0)	3 (3.4)	0 (0.0)	57 (6.9)	8 (7.8)	7 (7.4)	2 (6.3)
$60-79 K	96 (8.4)	8 (5.3)	8 (9.2)	4 (9.1)	73 (8.8)	9 (8.7)	12 (12.6)	1 (3.1)
$75-99 K	156 (13.6)	13 (8.7)	11 (12.6)	5 (11.4)	113 (13.6)	17 (16.5)	18 (18.9)	7 (21.9)
$100 K +	489 (42.6)	28 (18.7)	26 (29.9)	17 (38.6)	384 (46.3)	18 (17.5)	22 (22.2)	9 (28.1)
Unknown	136 (11.8)	19 (12.7)	12 (13.8)	13 (29.5)	84 (10.1)	19 (18.4)	14 (14.7)	8 (25.0)
**Housing, n (%)**								
Homeowner	814 (70.8)	86 (57.3)	47 (54.0)	24 (54.5)	633 (76.4)	56 (54.4)	64 (67.4)	15 (46.9)
Non-homeowner	102 (8.9)	29 (19.3)	20 (23.0)	2 (4.5)	66 (8.0)	16 (15.5)	11 (11.6)	6 (18.8)
Unknown	233 (20.3)	35 (23.3)	20 (23.0)	18 (40.9)	130 (15.7)	31 (30.1)	20 (21.1)	11 (34.4)
**Insurance type, n (%)**								
HMO	148 (12.9)	17 (11.3)	14 (16.1)	4 (9.1)	97 (11.7)	13 (12.6)	11 (11.6)	4 (12.5)
PPO	30 (2.6)	10 (6.7)	1 (1.1)	2 (4.5)	19 (2.3)	3 (2.9)	1 (1.1)	2 (6.3)
POS	755 (65.7)	89 (59.3)	52 (59.8)	35 (79.5)	526 (63.4)	61 (59.2)	53 (55.8)	20 (62.5)
Other	216 (18.8)	34 (22.7)	20 (23.0)	3 (6.8)	187 (22.6)	26 (25.2)	30 (31.6)	6 (18.8)
**QCI, mean (SD)**	0.6 (1.2)	0.7 (1.2)	0.4 (0.9)	0.5 (1.0)	0.7 (1.5)	1 (1.8)	0.5 (0.9)	0.6 (1.4)
**General comorbid conditions, n (%)**								
Abdominal pain	724 (63.0)	97 (64.7)	58 (66.7)	32 (72.7)	449 (54.2)	55 (53.4)	55 (57.9)	12 (25.5)
Diarrhoea	534 (46.5)	65 (43.3)	43 (49.4)	18 (40.9)	558 (67.3)	63 (61.2)	54 (56.8)	18 (38.3)
Anaemia	359 (31.2)	67 (44.7)	24 (27.6)	15 (34.1)	279 (33.7)	39 (37.9)	32 (33.7)	14 (29.8)
Nausea and vomiting	328 (28.5)	63 (42.0)	27 (31.0)	12 (27.3)	178 (21.5)	22 (21.4)	21 (22.1)	3 (6.4)
Hypertension	267 (23.2)	50 (33.3)	15 (17.2)	4 (9.1)	224 (27.0)	37 (35.9)	22 (23.2)	4 (8.5)
Anxiety	252 (21.9)	34 (22.7)	15 (17.2)	2 (4.5)	161 (19.4)	11 (10.7)	13 (13.7)	3 (6.4)
Hyperlipidemia	223 (19.4)	38 (25.3)	16 (18.4)	4 (9.1)	241 (29.1)	28 (27.2)	21 (22.1)	6 (12.8)
Depression	197 (17.1)	24 (16.0)	8 (9.2)	5 (11.4)	106 (12.8)	8 (7.8)	12 (12.6)	2 (4.3)
Bloating	112 (9.7)	14 (9.3)	12 (13.8)	4 (9.1)	68 (8.2)	6 (5.8)	10 (10.5)	2 (4.3)
Cardiovascular disease	94 (8.2)	14 (9.3)	7 (8.0)	1 (2.3)	63 (7.6)	11 (10.7)	8 (8.4)	0 (0.0)
Diabetes	87 (7.6)	17 (11.3)	8 (9.2)	1 (2.3)	85 (10.3)	16 (15.5)	10 (10.5)	2 (4.3)
**Immune-mediated inflammatory diseases, n (%)**								
Rheumatoid arthritis	29 (2.5)	7 (4.7)	4 (4.6)	0 (0.0)	22 (2.7)	4 (3.9)	1 (1.1)	0 (0.0)
Psoriatic arthritis	11 (1.0)	1 (0.7)	1 (1.1)	0 (0.0)	12 (1.4)	1 (1.0)	0 (0.0)	0 (0.0)
Psoriasis	38 (3.3)	4 (2.7)	1 (1.1)	0 (0.0)	21 (2.5)	2 (1.9)	0 (0.0)	0 (0.0)
**Surgery, n (%)**	181 (15.8)	21 (14.0)	18 (20.7)	6 (13.6)	38 (4.6)	10 (9.7)	6 (6.3)	4 (8.5)
**Medication use, n (%)**								
Opioids	594 (51.7)	81 (54.0)	45 (51.7)	16 (36.4)	345 (41.6)	42 (40.8)	29 (30.5)	14 (29.8)
Antibiotics	393 (34.2)	51 (34.0)	28 (32.2)	14 (31.8)	255 (30.8)	31 (30.1)	27 (28.4)	7 (14.9)
5-Aminosalicylates	258 (22.5)	37 (24.7)	27 (31.0)	11 (25.0)	580 (70.0)	72 (69.9)	72 (75.8)	23 (48.9)
Steroids	794 (69.1)	114 (76.0)	55 (63.2)	31 (70.5)	671 (80.9)	78 (75.7)	75 (78.9)	26 (55.3)
Immunomodulators	225 (19.6)	22 (14.7)	15 (17.2)	14 (31.8)	146 (17.6)	19 (18.4)	12 (12.6)	12 (25.5)

*CD* Crohn’s disease, *HMO* Health maintenance organization, *n* Number of patients, *POS* Point of service, *PPO* Preferred provider organization, *QCI* Quan-Charlson comorbidity index, *SD* Standard deviation

#### Treatment adherence and persistence for patients with CD

**Fig. 2:**
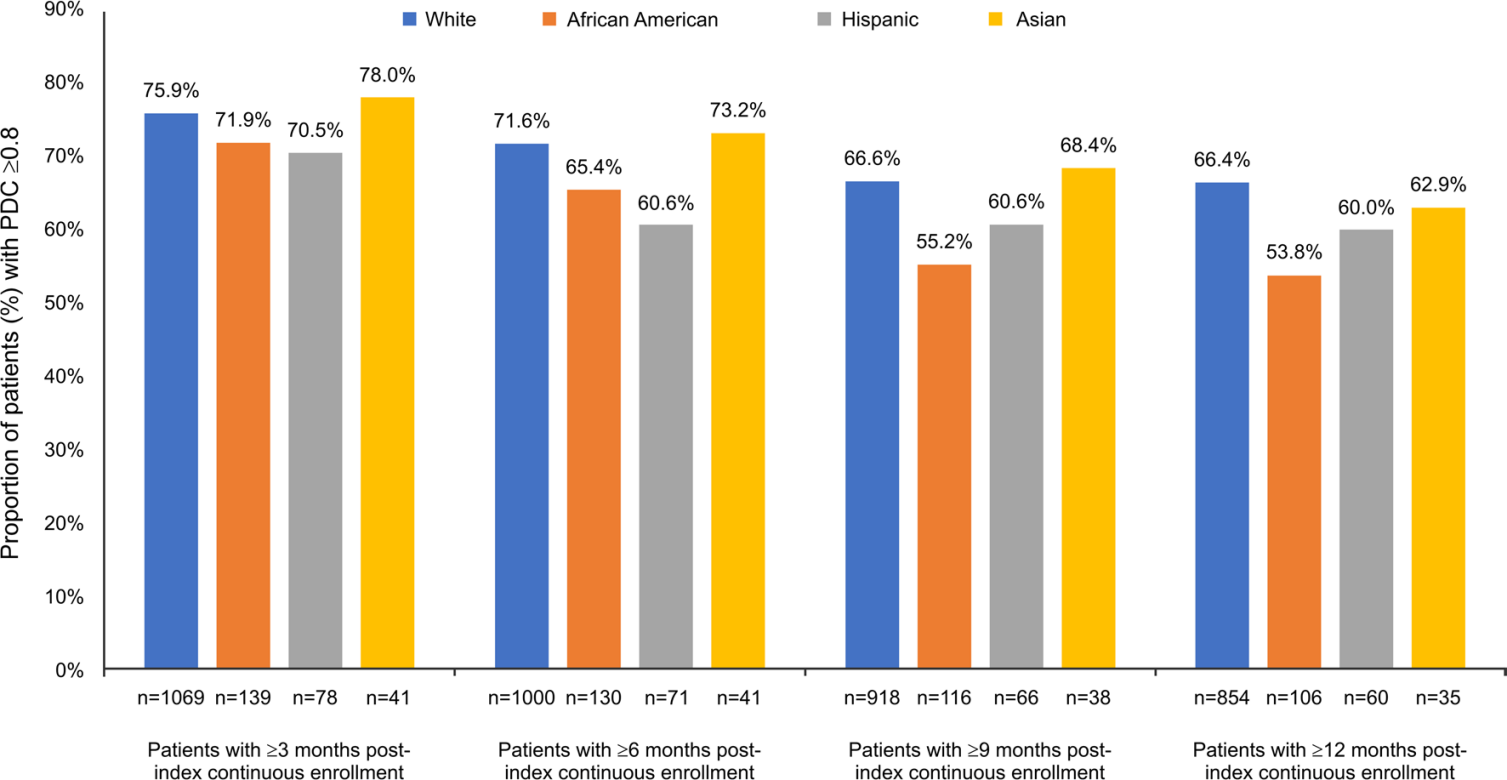
Treatment adherence for patients with CD. CD, Crohn’s disease; PDC, proportion of days covered

During the 12-month post-index period, 66.4% of White patients were adherent to their biologics, followed by Asians (62.9%), Hispanics (60.0%), and AA (53.8%). Overall, White patients had numerically better treatment adherence as compared with the other racial/ethnic groups (Fig. 2).

**Fig. 3:**
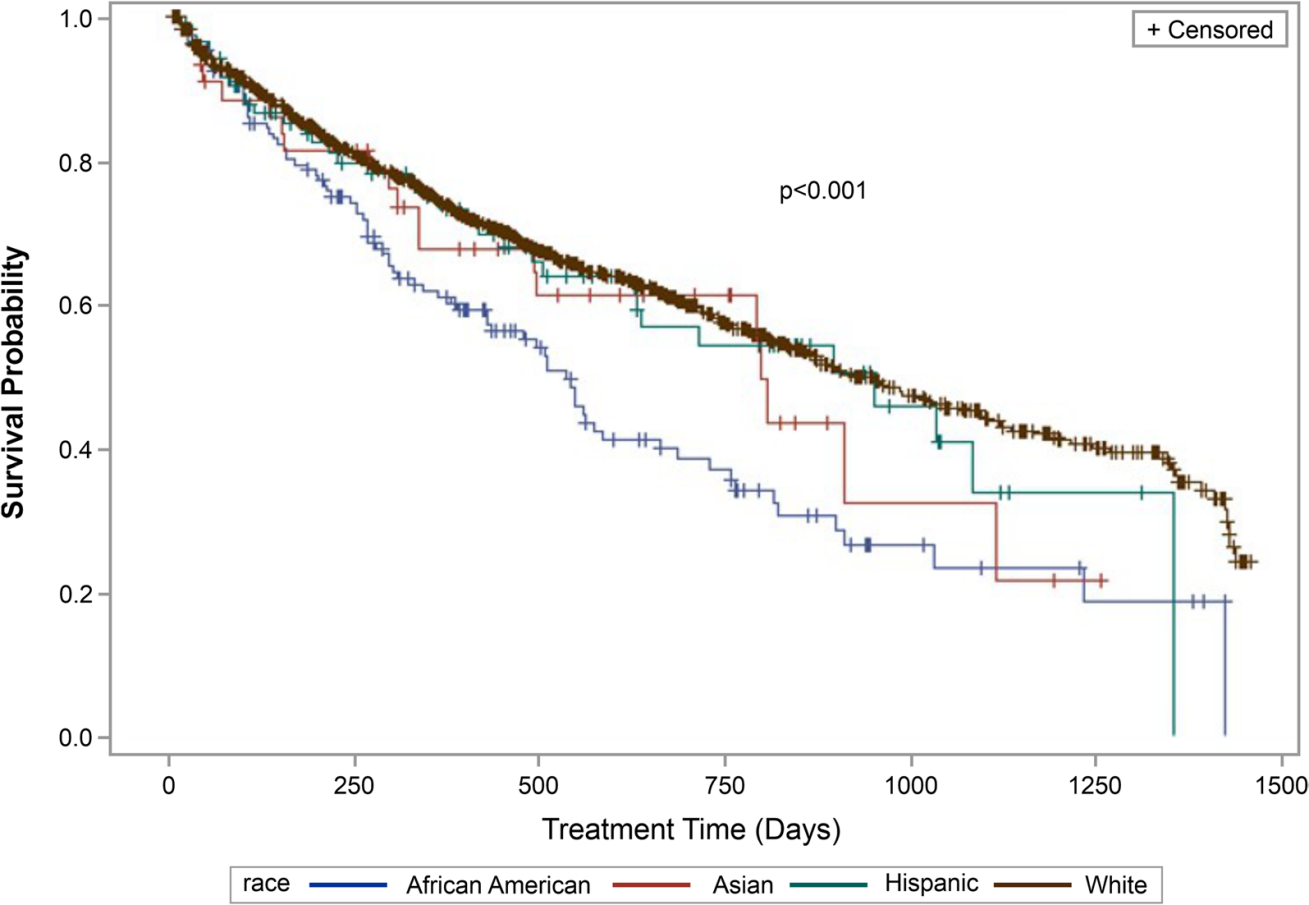
Kaplan-Meier analysis of time-to-treatment discontinuation (persistence) across racial groups for CD (*N* = 1,430) patients Note: Survival indicates probability of patients continuing treatment. CD, Crohn’s disease

A total of 40.8% of patients discontinued biologics at a median follow-up of 278 days. The percentage of AA discontinuing biologics were 54.7%, followed by Asians (45.5%), Hispanics (39.1%), and Whites (38.9%). The Kaplan–Meier curves indicated that AA had a significantly (*p* < 0.001) shorter time to treatment discontinuation as compared with other racial/ethnic groups (median days, AA: 533; White: 919; Hispanic: 948; Asian: 796) (Fig. 3).

Among patients with 12-month post-index continuous enrollment (*n* = 916), the adjusted multivariate regression analysis showed that AA patients were less likely to adhere to biologics during the 12-month period compared with White patients (adjusted OR [95% CI]: 0.61 [0.38; 0.99]). No significant difference in the treatment adherence was observed between White patients and other racial/ethnic groups after adjusting for other SES and clinical characteristics (Table 2).

**Table 2:** Multivariate analysis for treatment adherence and discontinuation in patients with CD or UC

Parameters	12-month Adherence		Time-to-discontinuation during follow-up	
	Unadjusted OR (95% CI)	Adjusted OR^a^ (95% CI)	Unadjusted HR (95% CI)	Adjusted HR^a^ (95% CI)
**CD**	***n* = 916**		***n* = 1250**	
African American vs White	0.55 (0.36; 0.86)	0.61 (0.38; 0.99)	1.75 (1.36; 2.26)	1.52 (1.16; 2.00)
Asian vs White	1.11 (0.48; 2.60)	0.97 (0.41; 2.32)	1.04 (0.60; 1.80)	1.25 (0.71; 2.20)
Hispanic vs White	0.70 (0.40; 1.23)	0.70 (0.39; 1.29)	1.15 (0.79; 1.67)	1.10 (0.75; 1.61)
**UC**	***n* = 682**		***n* = 934**	
African American vs White	1.20 (0.68; 2.14)	1.45 (0.78; 2.71)	0.99 (0.71; 1.39)	1.00 (0.71; 1.42)
Asian vs White	0.73 (0.29; 1.89)	0.56 (0.20; 1.56)	0.80 (0.41; 1.55)	0.83 (0.42; 1.63)
Hispanic vs White	0.93 (0.53; 1.64)	0.85 (0.47; 1.55)	1.00 (0.72; 1.39)	1.14 (0.81; 1.60)

*CD* Crohn’s disease, *CI* Confidence interval, *OR* Odds ratio, *HR* Hazard ratio, *UC* Ulcerative colitis

Multivariate analysis for treatment adherence and discontinuation was performed in patients with non-missing household income

^a^ Other variables adjusted in the regression models include baseline clinical and sociodemographic characteristics including age, sex, education level, income, insurance plan type, hypertension, nausea and vomiting, anemia, rheumatoid arthritis, psoriasis, psoriatic arthritis, surgery, opioid use, steroid use, immunomodulators

The adjusted Cox proportional-hazards model revealed that AA patients were more likely to discontinue biologics earlier during the follow-up period (adjusted HR [95% CI]: 1.52 [1.16; 2.0]) compared with White patients. Other racial/ethnic groups did not show significant differences compared with White patients (Table 2).

### Patients with UC

#### Socioeconomic status and clinical characteristics

Of the 1059 UC patients, more than half (53.2%) were male, and the mean (SD) age was 44.7 (17.2) years. The majority of patients were White (78.3%), followed by AA (9.7%), Hispanic (9.0%), and Asian (3.0%). Approximately three-fourths of the AA patients (75.7%) resided in the South region (Virginia, North Carolina, South Carolina, Georgia, Alabama, Mississippi), with low education (only 9.7% had a Bachelor’s degree and above) and low household income (23.3% had < $40 k income). The mean (SD) QCI was 0.7 (1.5), and the most frequently observed comorbid condition was diarrhea (65.4%), followed by abdominal pain (53.9%) and anemia (34.4%).

### Treatment adherence and persistence for patients with UC

**Fig. 4:**
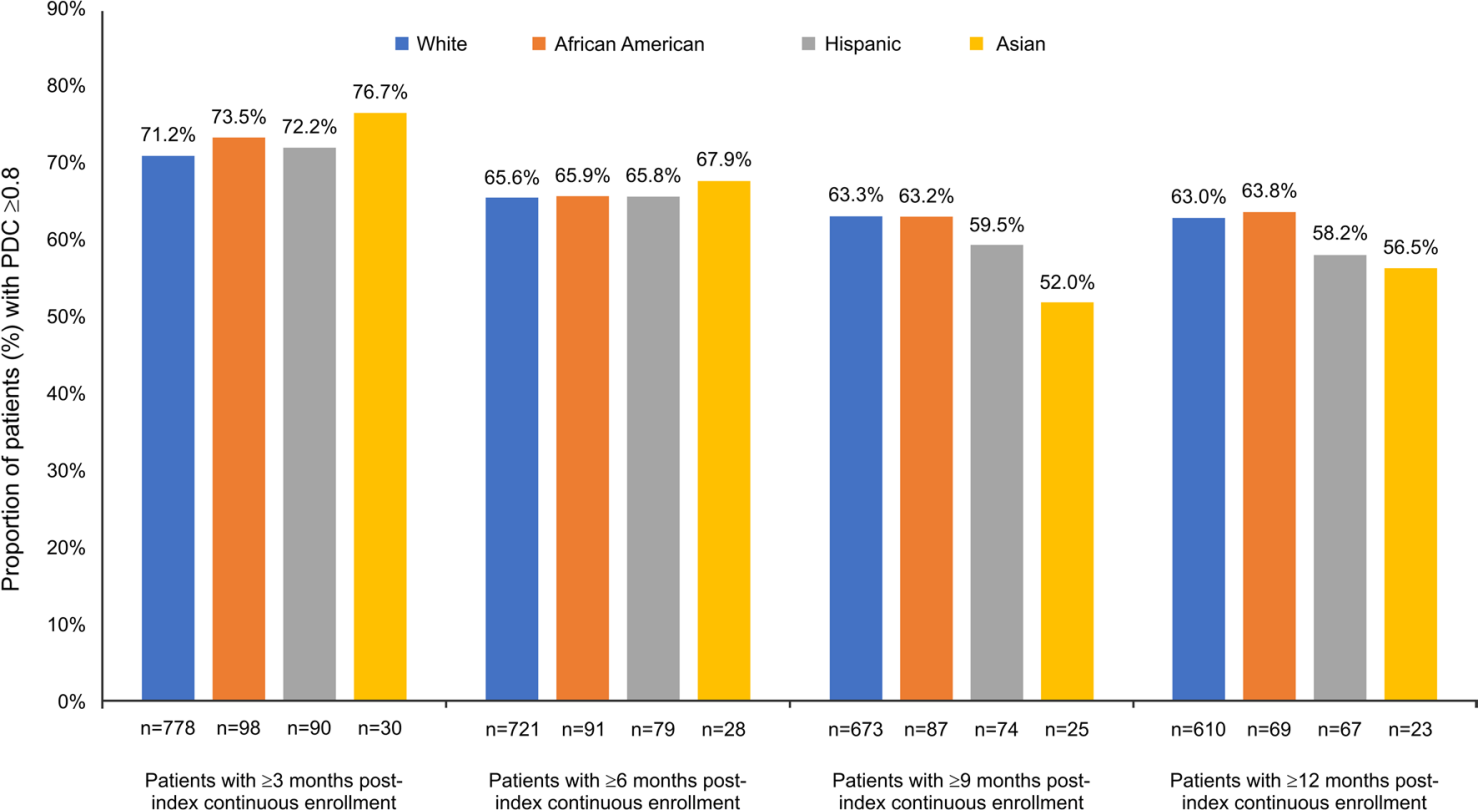
Treatment adherence for patients with UC. PDC, proportion of days covered; UC, ulcerative colitis

During the 12-month post-index period, 63.8% of AA patients were adherent to their biologics, followed by Whites (63%), Hispanics (58.2%), and Asians (56.5%). Figure 4 describes statistics on the treatment adherence for each racial/ethnic group of patients with different duration of continuous enrollment.

**Fig. 5:**
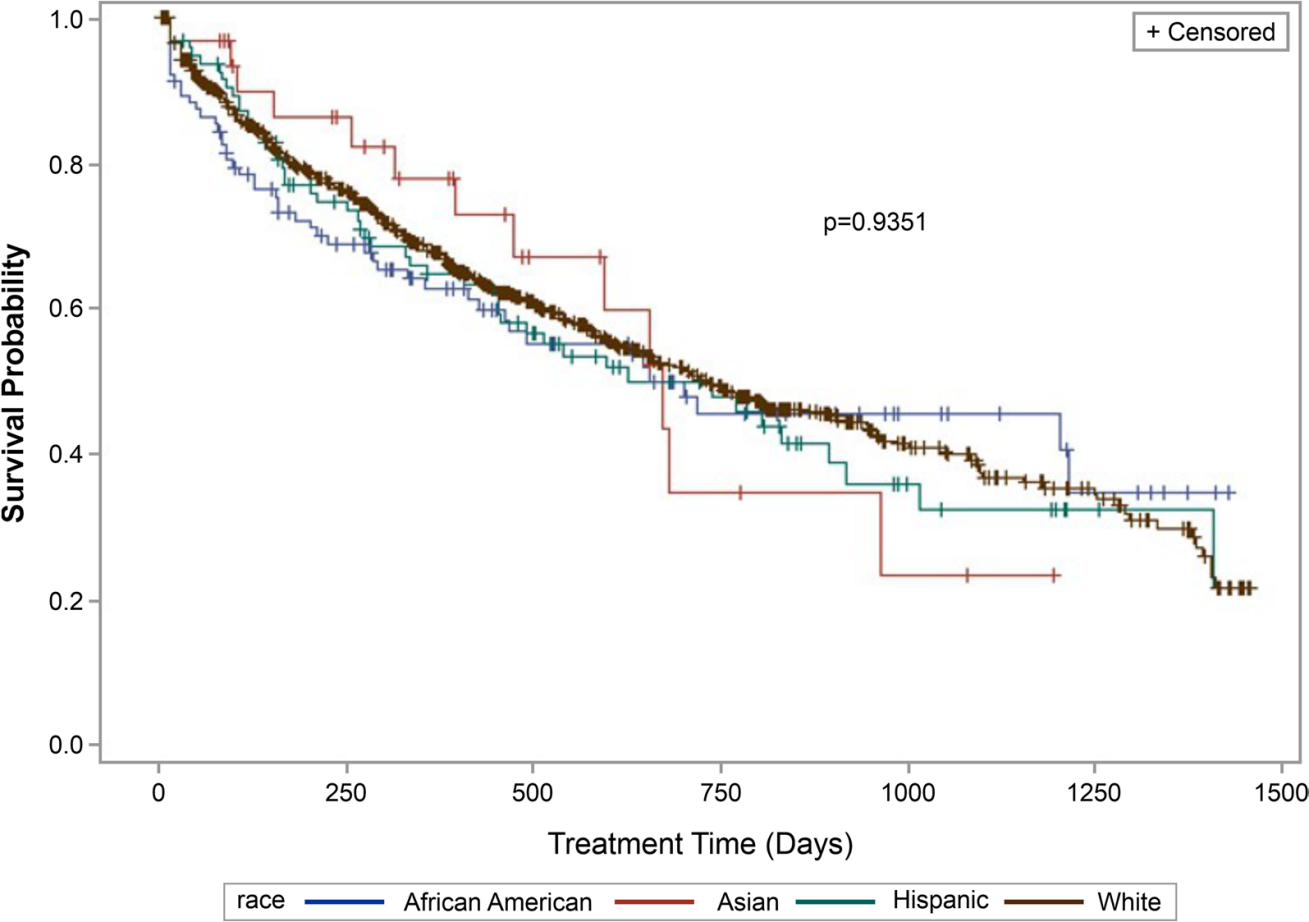
Kaplan Meier analysis of time-to-treatment discontinuation (persistence) across racial groups for UC (*N* = 1,059) patients Note: Survival indicates probability of patients continuing treatment. UC, ulcerative colitis

A total of 46.5% patients discontinued biologics at a median follow-up of 252 days. Numerically, Hispanic patients were the most likely to discontinue biologics whereas Asian patients were the least likely (White: 46.1%; AA: 46.6%; Hispanic: 51.6%; Asian: 40.6%). The Kaplan–Meier curves indicated no significant (*p* = 0.9351) differences in treatment persistence among the racial/ethnic groups (median days, AA: 701; White: 730; Hispanic: 626; Asian: 671) (Fig. 5).

Among patients with the 12-month post-index continuous enrollment (*n* = 682), the adjusted logistic regression analysis demonstrated that no statistically significant difference in the adherence to biologics for AA (adjusted OR [95% CI]: 1.45 [0.78; 2.71]), Asian (0.56 [0.20; 1.56]), and Hispanic (0.85 [0.47; 1.55]) patients as compared to White patients during the 12-month period (Table 2). Similarly, the adjusted Cox proportional-hazards regression analysis revealed no significant difference in the time to discontinuation of biologics for these racial/ethnic groups compared to White patients during the follow-up period (adjusted HR [95% CI]: AA: 1.00 [0.71; 1.42], Asian: 0.83 [0.42; 1.63], and Hispanic: 1.14 [0.81; 1.60]) (Table 2).

## Discussion

The use of biologic agents in the treatment of moderate-to-severe CD or UC has been well-established [21, 22]. It has been known that non‐adherence to biologics may affect disease remission, which in turn may lead to overall deteriorated health outcomes [25, 35, 36]. Furthermore, published literature have indicated that improving treatment adherence may be associated with overall improved disease status and decreased morbidity, mortality, and overall healthcare expenses [23–25]. However, limited evidence is available regarding the impact of race and ethnicity on treatment compliance and continuation among patients with IBD. This observational, retrospective study provides an important contribution to the existing literature, by assessing the association between race/ethnicity, treatment adherence, and persistence among patients with CD or UC who initiated biologics in a commercially insured population in the US.

Our study demonstrated a significant association between race/ethnicity, treatment adherence, and persistence for patients with CD even after controlling for socioeconomic and clinical characteristics. In our study, nearly two-thirds of the AA patients resided in the South region which was confirmed by the US 2020 Census (Census). More specifically, the Census showed that AA population are mostly concentrated in the South [37]. Compared to White patients, AA patients with CD in our study were less likely to adhere to biologics during the 12-month study period and more likely to discontinue biologics earlier. However, in patients with UC, no significant association between race/ethnicity, treatment adherence, or persistence was observed. The Asian population had the best adherence at month 3, 6, and 9, but not at month 12. In addition to the small sample size at month 12, the Asian population are culturally very different. It is possible that disease perceptions and acceptance of treatment are different in this culturally unique population. Previous studies of South Asian patients also reported lower rates of biologic use, suggesting that financial burden is unlikely to be the leading explanation for the lower use of biologic agents in South Asian patients. Asian patients were more likely to attribute their disease to “causes other than known causes,” which influenced their acceptance of treatment [38, 39]. Several studies have also reported racial differences in the adherence to biologics in patients with IBD. In a cross-sectional study by Nguyen et al., race was amongst the predictors of adherence with significantly higher adherence reported in Whites as compared with AA (80% vs. 50%; *P* < 0.0001) [40]. A systematic review published in 2013 evaluated 13 studies and reported a pooled adherence rate with biologics in patients with IBD at 82.6% (range: 36.8%-96%). Hispanic and AA were reported as predictors of non-adherence to biologics [41]. However, most of these studies evaluated the IBD population as a whole, and not patients with CD or UC separately [9, 40, 41]. Our study adds real-world evidence to the existing literature with a focus on the evaluation of biologic adherence and persistence for patients with CD or UC.

There is a lack of diversity in racial/ethnic populations in clinical trials among patients with IBD, with most of the patients being White and minorities underrepresented [19, 20]. Therefore, the implications on response to treatment and differences in the drug metabolism among different races may not be well understood. The current management approaches among patients with IBD are based on studies conducted primarily on White patients. Hence, clinical studies in diverse populations are warranted that may elicit a better understating of the IBD manifestations, possible treatment options, and their outcomes [42].

Our study has demonstrated racial differences in treatment adherence and persistence among patients with CD but not patients with UC. One of the possible reasons for such differential findings could be attributed to potential differences in disease manifestation/ treatment responses between CD and UC in patients’ ethnicities, particularly the most severe forms for e.g., the perianal and fistulizing disease in AA patients with CD compared with other races. However, consistent differences in the disease extent between different races/ethnicities in UC have not been demonstrated [42]. More severe disease would lead to better adherence to maximize the treatment effect, but if less effective, to greater discontinuation. It has also been suggested that penetration of biologics in UC is much less than CD. Findings from a large retrospective study with 9,145 biologic-naïve patients newly diagnosed with IBD from 2008 to 2015, included 5,612 patients (61.37%) with CD and 3,533 patients (38.63%) with UC suggested that patients with UC had slightly increased risk of stopping initial biologic treatment than patients with CD [43]. The clinical factors that may increase the risk of non-persistence, included having infection diagnosis in the past 30 days, changing diagnosis between CD and UC, treatment with steroids. In another similar retrospective study in 415,405 patients of which 188,842 patients with CD and 195,183 were patients with UC, during the 9-year study duration, the proportion of all patients using biologics increased in CD, 21.8% to 43.8%, whereas in UC, the proportion of patients using biologics increased from 5.1% to 16.2% [44]. Hospitalization, steroids use, and infection diagnosis are among the strongest predictors, suggesting treatment side effects and worsening disease are likely to be the major reason for discontinuation of treatment. A similar trend is observed in our study between adherence in patients with CD or UC. Additionally, different treatment practices in CD and UC can be a reason for discontinuation. Use of biologic is usually a late-line therapy in UC compared to CD, which is often viewed as “curable” after surgery, but CD is a chronic disease and warrants long-term therapy. There is underrepresentation of diverse groups in clinical trials and future studies are warranted for a better understanding of the attributes potentially associated with lack of adherence amongst minorities. Although genetics of CD in AA patients are well documented, under-representation in RCT persists in fully understanding therapeutic responses to treatment. Possible factors that lead to poor compliance in minority races require further investigation, particularly in patients with CD. Some of the factors that need to be looked into are racial differences in response to biologics treatment (genetic factors), the standard of care received by minorities compared to other races, delays in switching biologics treatments in the absence of clinical response in minorities, and providers’ perception regarding treatment management among different races [42]. Nearly all of the above factors are possible but with the possible exception of genetics, there shouldn’t be any difference in the other factors between CD or UC. Further studies are needed to better understand the possible reasons for observed racial differences in CD patients.

The present analysis has several limitations. First, the administrative claims data were collected to facilitate payment for healthcare services, and definitive diagnoses were not available. Second, the reasons for switching, discontinuation, dose escalation, or adding adjunctive therapy were not available, hence, the effect of these factors on treatment decisions/patterns was unknown. In patients with IBD, the loss of response to biologics is commonly encountered [45], and it has been demonstrated that patients’ response to the treatment affected the adherence or persistence [4]. In claims data, possible reasons for treatment discontinuation are not captured. Hence, an association of treatment response with adherence or persistence could not be assessed in our analysis. In addition, the use of adherence and persistence measures does not guarantee whether patients used their treatments as prescribed. Third, potential exists for a misclassification of race. Although SES variables are available in the database, some were collected directly from public records while the remaining data were imputed from algorithms using enhanced geocoding, predictive modeling, and the US Census data [46]. Last but not least, this study is limited to patients covered by commercial and Medicare Advantage; therefore, the results may not be generalizable to patients with other insurance types (such as Medicaid population with lower SES), or those without health insurance coverage.

## Conclusions

Findings from this study provide insights into the SES, clinical characteristics, treatment adherence, and persistence by race/ethnicity among patients with CD or UC initiated with biologics in the US. Compared to White patients, AA patients with CD were less likely to adhere to biologics, whereas in UC patients, no significant racial differences were observed. AA patients with CD were more likely to discontinue biologics earlier compared to White patients, whereas no significant racial differences were observed in patients with UC. Future large-scale studies in patients with CD or UC are warranted to validate the findings reported in this study, particularly to further understand possible factors leading to poor treatment compliance in patients with CD among diverse populations.

## Data Availability

The data sharing policy of Janssen Pharmaceutical Companies of Johnson & Johnson is available at https://www.janssen.com/clinical-trials/transparency. Requests for access to the study data can be submitted through Yale Open Data Access (YODA) Project site at http://yoda.yale.edu.

## Abbreviations

AA: African Americans
CD: Crohn's disease
CI: Confidence interval
FDA: Food and Drug Administration
HMO: Health maintenance organization
HR: Hazard ratio
IBD: Inflammatory bowel disease
LASSO: Least absolute shrinkage and selection operator
Optum: Optum Clinformatics
OR: Odds ratio
PDC: Proportion of days covered
PPO: Preferred provider organization
QCI: Quan-Charlson comorbidity index
RCT: Randomized controlled trials
SAS: Statistical analysis software
SES: Socioeconomic status
SD: Standard deviation
UC: Ulcerative colitis
US: United States
